# β‐TrCP overexpression enhances cisplatin sensitivity by depleting BRCA1

**DOI:** 10.1002/1878-0261.70089

**Published:** 2025-07-28

**Authors:** Rocío Jiménez‐Guerrero, Alejandro Belmonte‐Fernández, Mónica González‐Moreno, Laura Rodríguez‐Cordero, Begoña Pérez‐Valderrama, Joaquín Herrero‐Ruíz, Francisco Romero, Carmen Sáez, Miguel Á. Japón

**Affiliations:** ^1^ Instituto de Biomedicina de Sevilla (IBiS) Hospital Universitario Virgen del Rocío/CSIC/Universidad de Sevilla Spain; ^2^ Department of Microbiology, Faculty of Biology Universidad de Sevilla Spain; ^3^ Department of Oncology Hospital Universitario Virgen del Rocío Seville Spain; ^4^ Department of Pathology Hospital Universitario Virgen del Rocío Seville Spain

**Keywords:** BRCA1, cancer, cisplatin, DNA damage, drug resistance, β‐TrCP

## Abstract

Cisplatin is one of the most used anticancer chemotherapy agents; however, over time, patients develop resistance to the treatment, and survival rates drop dramatically. Investigation of tumor cell resistance mechanisms could increase sensitivity and prevent cancer progression. Here, we investigated the role of the E3 ubiquitin ligase SCF (β‐TrCP) in cisplatin resistance in different tumor cell lines, analyzing its role in the stability of BRCA1 and CtIP, proteins involved in DNA damage repair by homologous recombination. We showed that SCF(β‐TrCP) plays a key role in cisplatin response, as overexpression of wild‐type β‐TrCP increased DNA damage and cisplatin‐induced apoptosis, while overexpression of a dominant‐negative mutant or siRNA‐mediated downregulation of β‐TrCP decreased the damage and conferred treatment resistance. Furthermore, we demonstrated that BRCA1 and CtIP interacted with β‐TrCP *in vivo*, and their levels changed when β‐TrCP expression was modulated. We also described that β‐TrCP‐mediated BRCA1 degradation involves both lysosomal and proteasomal pathways. Mechanistically, the failure of β‐TrCP to regulate the degradation of BRCA1 enables a more efficient DNA damage repair and thereby the acquisition of cisplatin resistance. Overall, β‐TrCP overexpression sensitizes cisplatin‐induced DNA damage by depleting BRCA1.

## Graphical abstract

Low levels of β‐TrCP (Panel A) allow the accumulation of BRCA1 and CtIP, which facilitate the repair of cisplatin‐induced DNA damage via homologous recombination (HR) and promote tumor cell survival. In contrast, high β‐TrCP expression (Panel B) leads to BRCA1 and CtIP degradation, impairing HR repair, resulting in persistent DNA damage and apoptosis. Therefore, β‐TrCP emerges as a key regulator of cisplatin sensitivity.

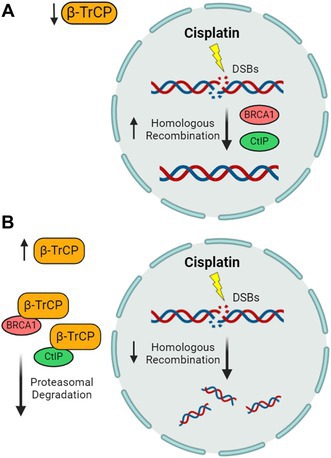

**Abbreviations**
ConA: concanamycin A
DMSO: dimethyl sulfoxide
DSBs: double‐strand breaks
HA: hemagglutinin
HR: homologous recombination
NHEJ: nonhomologous DNA end joining
siRNA: small interfering RNA
ssDNA: single‐stranded DNA
UPS: ubiquitin‐dependent proteasome system

## 1 Introduction

Cisplatin is widely used in the treatment of different types of cancer including cervical, ovarian, testicular, head and neck, breast, bladder, stomach, prostate, and lung cancers [1]. However, over time patients become resistant to the treatment and survival drops dramatically, so the study of the resistance mechanisms of tumor cells can help to develop new strategies to increase sensitivity and prevent cancer progression. Cisplatin creates bonds between the two DNA strands by covalently binding to purine DNA bases, inducing DNA double‐strand breaks (DSBs), interfering with DNA repair mechanisms and, subsequently, inducing apoptosis in cancer cells [1, 2, 3]. Increased DNA repair is considered the most significant characteristic of cisplatin‐resistant cells [4]. Eukaryotic cells use two main repair mechanisms for DSBs, homologous recombination (HR) and nonhomologous DNA end joining (NHEJ), as well as several secondary pathways. While the NHEJ mechanism is active during interphase, HR occurs mainly after replication in the S and G2 phases of the cell cycle, when an identical sister chromatid is available as a template for repair, so this mechanism, unlike the other, is generally error‐free. The signaling pathway of the HR system is executed by sequential recruitment of several nucleases that catalyze the resection of DNA broken ends to generate extended regions of single‐stranded DNA (ssDNA). The primary sensor of DSBs is the highly conserved MRN complex (MRE11‐RAD50‐NBS1), which binds to both sides of the breaks allowing CtIP‐mediated resection. Subsequently, the accumulation of numerous proteins occurs in a complex mechanism that employs phosphorylation mediated by kinases, including BRCA1/2, RAD51, and ubiquitination mediated by E3‐ubiquitin ligases. In this way, the resulting RAD51‐ssDNA complex then performs the homology search that allows DNA repair to complete the process and restore the chromosomes [5, 6]. Thus, while maintaining the repair machinery intact is critical to prevent DNA damage and tumor processes, the integrity of these pathways can also be detrimental to cancer treatment, and there is extensive preclinical evidence suggesting how alterations in DNA repair mechanisms are associated with sensitivity or resistance to cisplatin chemotherapy [7, 8]. Degradation pathways are important for cellular homeostasis and maintenance of the repair machinery. The two major protein degradation pathways in eukaryotic cells are the autophagy/lysosome pathway and the ubiquitin‐dependent proteasome system (UPS). Both pathways have been shown to play an essential role in tumorigenesis, and the interest in developing therapeutic agents that inhibit or enhance protein degradation is steadily growing [9]. The degradation of proteins by the UPS takes place if they are previously ubiquitinated, which occurs by a cascade of reactions involving at least three enzymes: E1 ubiquitin‐activating enzyme that activates the ubiquitin molecule, E2 ubiquitin‐conjugating enzyme that transfers ubiquitin, and E3 ubiquitin‐protein ligase enzyme that finally binds the ubiquitin molecule to the substrate protein [10, 11, 12]. One of the main E3 enzymes is the ubiquitin ligase SKP1‐CUL1‐F‐box SCF(β‐TrCP), which recognizes a wide range of proteins with antagonistic roles in tumorigenesis so that it can behave as oncogenic or as a tumor suppressor depending on the cellular context [13]. For example, β‐TrCP overexpression correlates with poor prognosis in tumors, such as colorectal cancer [14] or hepatoblastoma [15]. This oncogenic function could be explained through the degradation of certain known substrates, such as IκB, a negative regulator of NFκB signaling, or CHK1 after DNA damage, whose degradation allows cells to continue cycling, leading to genomic instability and tumor progression. However, somatic inactivating mutations of β‐TrCP have been identified in various human neoplasms, such as gastric, prostate, or breast cancer that would facilitate the activation of the Wnt signaling pathway by stabilizing its substrate β‐catenin, thus promoting tumor development [16, 17, 18]. Proteins involved in DSBs repair are regulated by ubiquitination at multiple levels [5], and it has also been shown that β‐TrCP could be involved in drug resistance in different tumors [19]. The molecular mechanism underlying these remains to be elucidated; however, considering that a single E3 ubiquitin ligase targets multiple substrates, as well as the specific cellular context of each case, SCF(β‐TrCP) could become a potential therapeutic target. Therefore, in this study, we have investigated the role of SCF(β‐TrCP) in the cisplatin resistance in different tumor cells, analyzing its influence on the regulation of proteins involved in DNA damage repair by HR, such as BRCA1 or CtIP.

## 2 Materials and methods

### 2.1 Cell culture and drugs

HT1376 (RRID:CVCL_C3H1), HT1197, and SKOV3 cell lines (RRID:CVCL_0532) were ordered from Sigma‐Aldrich (St. Louis, MO, USA). U2OS (RRID:CVCL_0042), HeLa (RRID:CVCL_0030), and Cos‐7 (RRID:CVCL_0224) cell lines were from the American Type Culture Collection (Manassas, VA, USA). 5637 and T47D (RRID:CVCL_0553) cell lines were ordered from the Interlab Cell Line Collection (Genoa, Italy). HT1197 cell line was recently purchased, while Cos‐7, U2OS, HeLa, T47D, 5637, and HT1376 cell lines were recently authenticated by STR profiling. Routinely, all the cells were cultured in MEM/EBSS (HyClone, Logan, UT, USA) supplemented with 10% fetal bovine serum (Biowest, Nuaillé, France), 50 U·mL^−1^ penicillin, 50 mm streptomycin, and 1 mm MEM nonessential amino acid solution (Sigma‐Aldrich). Cells were cultured at 37 °C in a humidified incubator under 5% CO_2_. All experiments were performed using cells that had not exceeded the first 10 passages after receipt and were routinely tested and free from *Mycoplasma* contamination. Stock solution of cisplatin (Selleck, Houston, TX, USA) was prepared at 1 mm in H_2_O and stored at −20 °C. Stock solutions of Z‐VAD‐FMK (Selleck), MG132 (Sigma‐Aldrich), rapamycin (Sigma‐Aldrich), and concanamycin A (ConA, Sigma‐Aldrich) were prepared at 10 mm in dimethyl sulfoxide (DMSO; Sigma‐Aldrich) and stored at −20 °C. In all experiments, cells were treated with either drug or vehicle during the log phase of growth. Cells were treated with 5 μm cisplatin, 20 μm ZVAD‐FMK, 20 μm MG132, 1–10 μm rapamycin, or 50 nm ConA when indicated.

### 2.2 Plasmid and small interfering RNA (siRNA) transfections

pCS2HA‐βTrCP and pCS2HA‐βTrCP ∆F plasmids have been previously described [20]. Cells were stably transfected using the lentivirus infection method as follows. Cells were seeded in 6‐well plates (Thermo Fisher Nunc, Roskilde, Denmark) and treated with 10 μL virus diluted in 1 mL medium/well when they were subconfluent. After 24 h, the medium was changed and 20 μg/mL blasticidin (Invivogen, San Diego, CA, USA) was added to isolate clones with the transfected cells. Finally, viable cells were cultured without blasticidin and amplified for freezing. For transient silencing of β‐TrCP1/2, we used the oligonucleotides 5′‐AAGUGGAAUUUGUGGAACAUC‐3′ and 5′‐GAUGUUCCACAAAUUCCACUUACUU‐3′ (Sigma‐Aldrich). For transient silencing of BRCA1, we used validated ON‐TARGET plus SMART pool L‐003482 (Dharmacon, Lafayette, CO, USA). ON‐TARGET plus SMART pool nontargeting (D‐001810, Dharmacon) siRNA was used as a negative control. The silencing efficiency provided by the manufacturer is 70%. Transfections were carried out using DharmaFECT 2 reagent (Dharmacon) according to the manufacturer's instructions. siRNA pools were used at 50 nm. The cells were subjected to the different treatments 24 h after the siRNA transfections were performed.

### 2.3 Cell viability assay

Alamar Blue Cell Viability Assay (Thermo Fisher Invitrogen, Carlsbad, CA, USA) was used according to the manufacturer's instructions. Briefly, 15 000 cells in exponential cell growth were seeded in 96‐well plates (Thermo Fisher Nunc) and the cells were treated with serial dilutions of cisplatin to span the dose range suitable from 0.05 to 50 μm during 48 h in triplicates. Fluorescence was measured with CLARIOstar (BMG Labtech, Ortenberg, Germany), and the data were normalized to the vehicle treatment using graphpad prism, RRID:SCR_002798 (GraphPad, San Diego, CA, USA).

### 2.4 Clonogenicity assay

To measure the ability to form individual clones after cisplatin treatment, 10 000 cells were plated in 6‐well plates in triplicates. After cell adherence, cells were treated with 5 μm cisplatin for 48 h and then the drug was removed. The numbers of colonies were counted after 15 days (where a colony is a group of cells consisting of 10 or more cells) using imagej (RRID:SCR_003070) (http://imagej.nih.gov/ij/). The cells were fixed and stained using either a Quick Panoptic solution (QCA, Tarragona, Spain) or a 0.5% solution of crystal violet in 20% methanol. Images were visualized on a confocal microscope (Leica THUNDER, Wetzlar, Germany) at 4× magnification.

### 2.5 Reverse transcription quantitative PCR

Total RNA was extracted from cells and purified using the AllPrepR DNA/RNA/miRNA Universal Kit (Qiagen, Hilden, Germany), and reverse transcription was performed with 1 μg of mRNA using the First Strand cDNA Synthesis Kit (Roche, Mannheim, Germany) according to the manufacturer's instructions. Quantitative PCRs were performed in triplicates in a volume of 25 μL using the SensiFAST™ SYBR No‐ROX Kit (BioLine, London, UK) according to the manufacturer's instructions. The specific primer oligonucleotides were designed with the Primer3 tool (RRID:SCR_003139) (https://primer3.ut.ee/) and manufactured at Sigma‐Aldrich: β‐TrCP (forward 5′‐CATTGTTTCTGCATCTGGGGAT‐3′ and reverse 5′‐TCAAATCGAATACAACGCACCA‐3′) and HPRT1 (forward 5′‐CGTCTTGCTCGAGATGTGAT‐3′ and reverse 5′‐GAGCACACAGAGGGCTACAA‐3′). PCR reaction was performed on a SmartCycler II real‐time PCR system (Cepheid, Sunnyvale, CA, USA) according to the following conditions: 150 s at 95 °C, followed by 40 cycles of 15 s at 95 °C, 30 s at 60 °C, and 30 s at 72 °C. The expression of the analyzed genes was calculated by applying the 2^−ΔCt^ relative quantification method, using the HPRT1 gene as an endogenous control. Standard curves were generated prior to qPCR. Different concentrations of cDNA template were used to construct the efficiency curve.

### 2.6 Tandem mass spectrometry (MS/MS)

pCS2HA‐β‐TrCP was transfected into Cos‐7 cells. After 18 h, cells were treated for 4 h with the calpain and proteasome inhibitor Ac‐LLnL‐CHO (Sigma‐Aldrich) and HA‐βTrCP immunoprecipitated from nuclear and cytosolic fractions with anti‐HA (BioLegend) previously coupled to AminoLink Plus Coupling Resin (Thermo Fisher Pierce, Rockford, IL, USA). Resins were washed three times in NP‐40 lysis buffer and six times in 50 mm ammonium bicarbonate. Samples were analyzed by MS/MS using an LTQ mass spectrometer (Thermo Scientific, Waltham, MA, USA) as described previously [21].

### 2.7 Antibodies

Mouse monoclonal anti‐PARP (1 : 750 #551024, RRID:AB_394008) and anti‐β‐catenin (1 : 10 000, #610153) were from BD Biosciences (San Jose, CA, USA); mouse monoclonal anti‐CtIP (1 : 1000, #sc‐271339, RRID:AB_10608728), anti‐CDK1 (1 : 4000, #sc‐54, RRID:AB_627224), and anti‐actinin (1 : 500, #sc‐17829, RRID:AB_626633) and anti‐RAD51 (1 : 50, #sc‐53428, RRID:AB_630180) were from Santa Cruz Biotechnology (Dallas, TX, USA); mouse monoclonal anti‐β‐actin (1 : 20 000, #A5441, RRID:AB_476744), anti‐α‐tubulin (1 : 20 000, #T9026, RRID:AB_477593), rabbit polyclonal anti‐LC3B (1 : 2000, #L7543, RRID:AB_796155), and rabbit polyclonal anti‐P62/SQSTM1 (1 : 2000, #SAB5701338) were from Sigma‐Aldrich; rabbit polyclonal anti‐cleaved caspase‐3 (Asp175) (1 : 750, #9664, RRID:AB_2070042), anti‐Wee1 (1 : 1000, #4936, RRID:AB_2288509), and anti‐β‐TrCP (1 : 500, #4394, RRID:AB_10545763) were from Cell Signaling (Danvers, MA, USA); mouse monoclonal anti‐hemagglutinin (HA)‐peroxidase (1 : 3000, #12013819001, Roche Cat# 12013819001, RRID:AB_390917) was from Roche; rabbit polyclonal anti‐BRCA1 (1 : 2000, #ab191042, RRID:AB_2650501) was from Abcam (Cambridge, UK); rabbit policlonal anti‐Mcl‐1 (1 : 1000, #ADI‐AAP‐240, RRID:AB_2039361) was from Enzo (Farmingdale, NY, USA); mouse monoclonal anti‐γH2AX^Ser139^ (1 : 5000, #05‐636, RRID:AB_309864) and anti‐PLK1 (1 : 5000, #05‐844, RRID:AB_310836) were from Merck‐Millipore (Burlington, MA, USA).

### 2.8 Western blots

Cell lysis was performed in RIPA lysis buffer [50 mm Tris–HCl (pH 7.5), 150 mm NaCl, 10% glycerol, 1% SDS, 0.5% deoxycholyc acid and 1% NP40]. Equivalent amounts of total protein, as determined by the BCA Protein Assay (Thermo Fisher Pierce), were subjected to SDS/PAGE on 6–10% polyacrylamide gels and transferred to Hybond ECL nitrocellulose membranes (GE Healthcare, Little Chalfont, UK). To ensure equal amounts of protein, the blots were stained with Ponceau S. For immunodetection, blots were soaked in blocking buffer [1% Blocking Reagent (Roche) in 0.05% Tween 20‐PBS] for 1 h and incubated with primary antibody in blocking buffer overnight at 4 °C. They were then washed in 0.05% Tween 20‐PBS and incubated with either goat anti‐mouse IgG (1 : 20 000, # NA931, RRID:AB_772210; GE Healthcare) or donkey anti‐rabbit IgG (1 : 20 000, #NA934V; GE Healthcare) peroxidase‐labeled antibodies in blocking buffer for 1 h. ECL™ Prime or ECL™ Select (GE Healthcare) was applied according to the manufacturer's protocol. The experiments were performed in triplicates unless otherwise specified. Blots were scanned using the imagelab software (Bio‐Rad, Hercules, CA, USA).

### 2.9 Co‐immunoprecipitation

Whole‐cell extracts (1–2 mg) diluted in 150 mm NaCl were incubated with normal rabbit serum (Santa Cruz) for 30 min and then with protein A sepharose beads (GE Healthcare) for 1 h at 4 °C. After centrifugation, the beads were discarded and the supernatants were incubated with either anti‐β‐TrCP rabbit polyclonal antibody or normal rabbit serum as a negative control overnight followed by protein A sepharose beads for 1 h. Beads were washed and bound proteins were solubilized by the addition of SDS sample buffer heated at 95 °C for 5 min.

### 2.10 Immunofluorescence

Immunofluorescence experiments were performed on cell culture 8‐chamber slides (BD). After the indicated treatment, cells were washed with PBS, fixed with paraformaldehyde for 20 min at room temperature, and permeabilized with 0.25% Triton (Sigma‐Aldrich) for 15 min at room temperature. Then, cells were washed with PBS again and blocked for 1 h with blocking buffer followed by overnight incubation at 4 °C with the primary antibody (1 : 500 γH2AX, RRID:AB_309864; Merck‐Millipore, and 1 : 50 RAD51, RRID:AB_630180; Santa Cruz). Then, cells were incubated with anti‐mouse IgG‐FITC (1 : 200, #F2012; Sigma‐Aldrich) for 2 h at room temperature, and nuclei were counterstained with 300 μm DAPI (Thermo Fisher Invitrogen) for 15 min at room temperature. Finally, cells were washed again with PBS and H_2_O and mounted with Fluorescent Mounting Medium (Agilent Dako, Glostrup, Denmark). Images were visualized on a confocal fluorescence microscope (Leica THUNDER, Wetzlar, Germany) at 40× magnification, and the foci of each nucleus were counted with software, imagej (RRID:SCR_003070) (http://imagej.nih.gov/ij/), using at least 100 cells of each condition from three independent experiments.

### 2.11 Cell cycle analysis

One million cells were trypsinized and fixed in 70% ethanol overnight. Cells were incubated for 1 h at 37 °C with RNase A solution 0.2 mg·mL^−1^ (Sigma). They were then stained with 20 μg·mL^−1^ propidium iodide solution (Sigma) for 15 min at 4 °C in the dark, and the DNA content was measured using the CellQuest Pro software on a FACScan flow cytometer (BD Biosciences). We quantified the percentage of cells in each cell cycle phase using the modfit lt2 software (Verity Software, Topsham, ME, USA).

### 2.12 Annexin V binding assays

Cells were exposed to the indicated treatments for 48 h. Subsequently, 1 × 10^6^ cells were harvested, washed in cold PBS, and resuspended in 100 μL of Annexin V binding buffer supplemented with 5 μL of propidium iodide and 5 μL of Annexin V‐FITC (Annexin V‐FITC Apoptosis Detection Kit I; BD Biosciences, # 556419). Samples were incubated for 15 min at room temperature in the dark, followed by the addition of 400 μL of binding buffer. Fluorescence was analyzed within 1 h using a FACScan flow cytometer (BD Biosciences). Apoptotic and viable cell populations were discriminated based on FL1 and FL2 fluorescence. The experiments were independently repeated three times, and data were processed using the cellquest pro software. A similar method has been previously described in published literature [22].

### 2.13 Statistical analysis

Experiments were performed in triplicates. Data comparing differences between two conditions were expressed as mean ± SD and statistically analyzed, when indicated, using two‐sided, paired Student's *t*‐test. Differences were considered significant when *P* < 0.05. Calculations were performed using prism 6.0 (GraphPad Prism, RRID:SCR_002798).

## 3 Results

### 3.1 β‐TrCP is involved in the response to cisplatin treatment

**Fig. 1:**
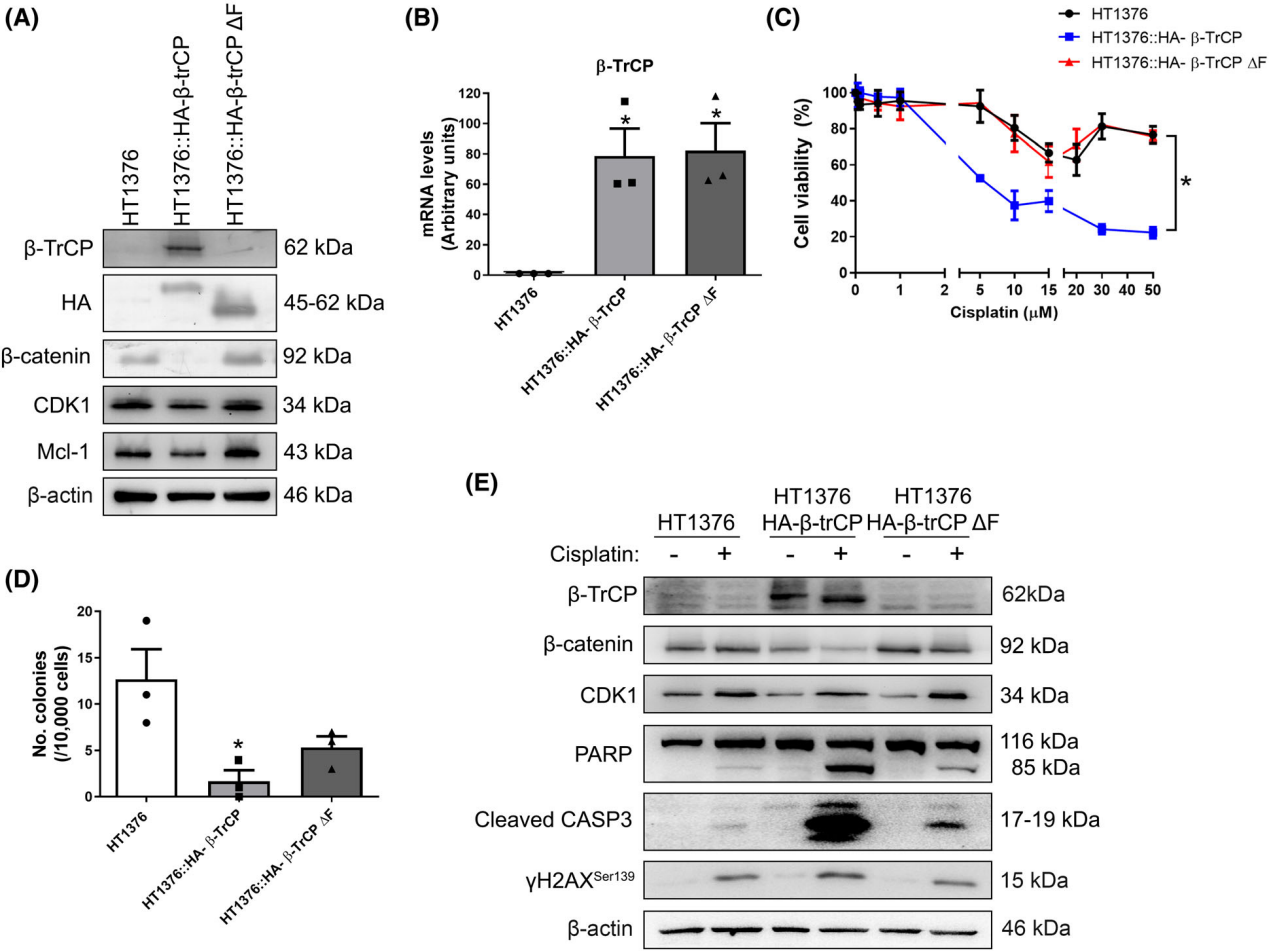
β‐TrCP modulates the response to cisplatin treatment. (A) Overexpression of wild‐type (HT1376::H‐β‐TrCP) and mutant (HT1376::HA‐β‐TrCP ΔF) forms of β‐TrCP in HT1376 cells. Western blots of β‐TrCP, hemagglutinin (HA), β‐catenin, CDK1, and Mcl‐1 are shown, using β‐actin as a loading control. (B) Expression levels of the β‐TrCP mRNA in the same cell lines. Histograms show quantification of mRNA levels by RT‐qPCR, using the HPRT1 gene as an endogenous control. (C) Cell viability assay for cisplatin in HT1376, HT1376::HA‐β‐TrCP, and HT1376::HA‐β‐TrCP ΔF cells. (D) Clonogenicity assay for cisplatin in HT1376, HT1376::HA‐β‐TrCP, and HT1376::HA‐β‐TrCP ΔF cells. They were treated with 5 μm cisplatin for 48 h. Histograms show the number of colonies formed after 15 days. (E) Western blots of β‐TrCP, β‐catenin, CDK1, PARP, cleaved caspase‐3, and γH2AX^Ser139^ in HT1376, HT1376::HA‐β‐TrCP, and HT1376::HA‐β‐TrCP ΔF treated with DMSO (vehicle) or 5 μm cisplatin for 48 h. β‐actin was used as a loading control. In all figures, data are presented as mean ± SD (standard deviation). *P value < 0.05 from Student's *t*‐test (All experiments were replicated three times).

To investigate the role of β‐TrCP in the cisplatin response, we firstly generated two stable cell lines from the HT1376 bladder cancer cells using lentivirus, one overexpressing the wild‐type β‐TrCP and the other overexpressing a dominant‐negative mutant of β‐TrCP, both tagged with hemagglutinin (HA), HT1376::HA‐β‐TrCP and HT1376::HA‐β‐TrCP ΔF, respectively. The ΔF mutation involves a deletion affecting the F‐box domain, which is required for the association with the SCF complex. As a result, while β‐TrCP ΔF can still interact with its substrates, it cannot promote their ubiquitination and degradation, acting as a dominant‐negative mutant. After verifying β‐TrCP or β‐TrCP ΔF overexpression at both protein (Fig. 1A and Fig. S1A) and mRNA levels (Fig. 1B), we observed a decrease in the levels of some of its substrates, such as β‐catenin, CDK1, or Mcl‐1 with the overexpression of the wild‐type β‐TrCP, while overexpression of the mutant β‐TrCP ΔF did not change their levels compared with the parental HT1376 cells (Fig. 1A). Interestingly, it is worth noting that the deletion also affected the region recognized by the β‐TrCP antibody. As a result, no β‐TrCP band was observed in the western blot for the HT1376::HA‐β‐TrCP ΔF cell line. However, both proteins were correctly detected using the HA antibody (Fig. 1A), as well as their mRNA by RT‐qPCR (Fig. 1B). Next, we carried out a viability assay with increasing concentrations of cisplatin. As we can see in Fig. 1C, in the wild‐type β‐TrCP‐overexpressing cells, there was a significant decrease in viability in comparison with the parental HT1376 cells.

This was further confirmed by a clonogenicity assay, in which cells were treated with 5 μm cisplatin for 48 h. After drug withdrawal, the cells were allowed to grow for 14 days to form colonies. As shown in Fig. 1D, wild‐type β‐TrCP‐overexpressing cells formed fewer colonies compared with parental HT1376 cells. However, in β‐TrCP ΔF‐overexpressing mutant cells (Fig. 1C,D), the phenotype more closely resembled control conditions, with viability similar to parental HT1376 cells but a less pronounced decrease in colony formation. This indicates that, although β‐TrCP‐ΔF cells resist cisplatin treatment, they do not fully return to a baseline state.

We also treated these three cell lines with 5 μm cisplatin for 48 h and we observed by western blot an increased apoptosis in wild‐type β‐TrCP‐overexpressing cells, as we can see by the levels of cleaved PARP and caspase‐3. This apoptosis was accompanied by DNA damage, indicated by higher levels of the DSB marker γH2AX^Ser139^. In contrast, when the mutant β‐TrCP ΔF was overexpressed, this effect was reversed and these markers decreased again (Fig. 1E and Fig. S1B). Apoptotic levels in all three lines after cisplatin treatment were also concordant with apoptosis analysis by Annexin V assay (Fig. S1C). Taken together, these results suggested that β‐TrCP is involved in the cisplatin response and that overexpression of this protein enhances the apoptotic response due to defects in the response to DNA damage.

### 3.2 Downregulation of β‐TrCP induces cisplatin resistance

**Fig. 2:**
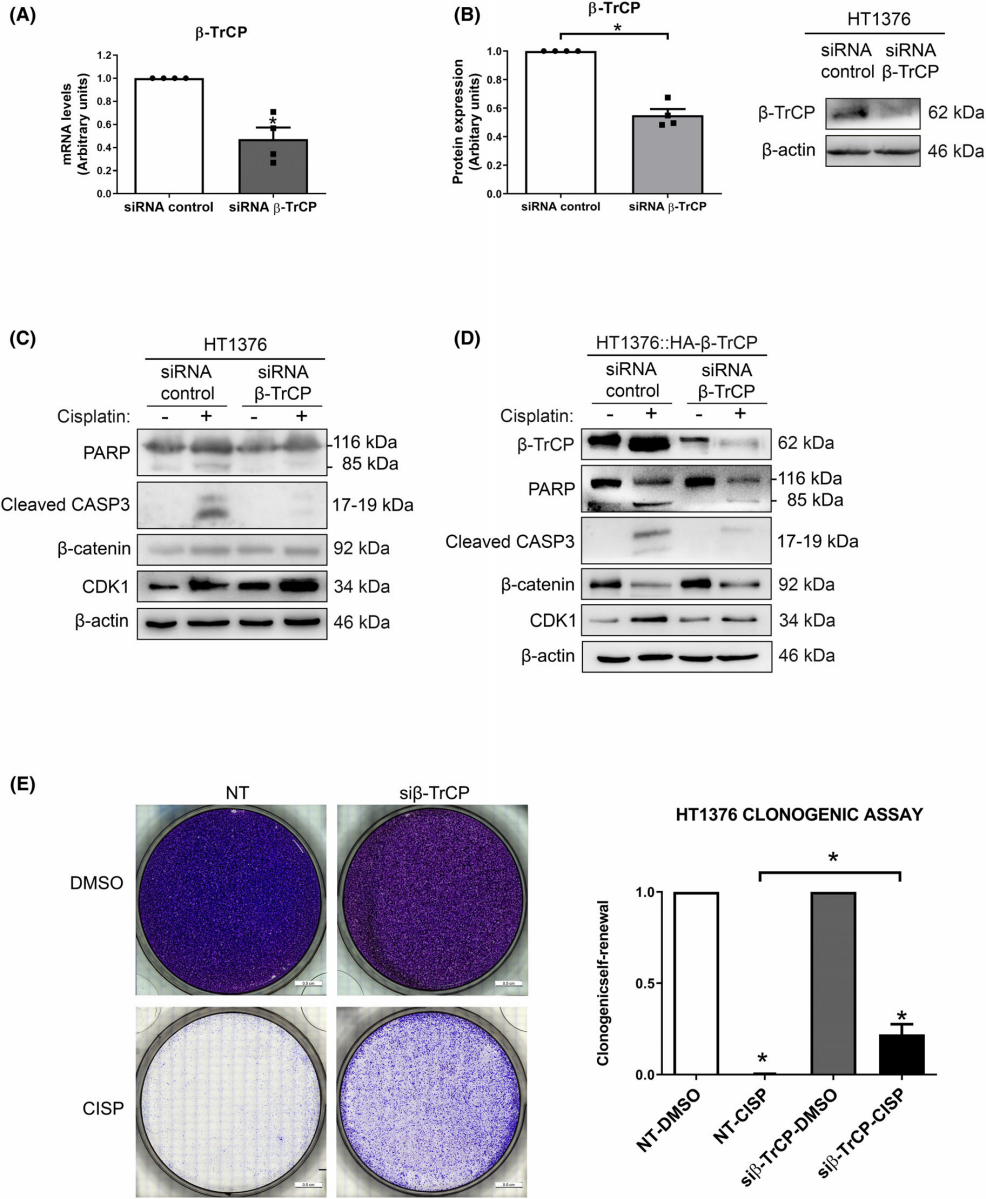
β‐TrCP gene silencing induces cisplatin resistance. (A) Expression levels of the β‐TrCP mRNA in HT1376 cells transfected with siRNA β‐TrCP or nontargeting siRNA. Histograms show quantification of mRNA levels by RT‐qPCR, using the HPRT1 gene as endogenous control. Data are presented as mean ± SD. *P value < 0.05 from Student's *t*‐test (All experiments were replicated three times). (B) Expression levels of β‐TrCP protein in the same conditions. Western blots of β‐TrCP, PARP, cleaved caspase‐3, β‐catenin, and CDK1 in (C) HT1376 and (D) HT1376::HA‐β‐TrCP cells transfected with siRNA β‐TrCP or nontargeting siRNA and treated with DMSO (vehicle) or 5 μm cisplatin during 48 h. β‐actin was used as loading control. (E) Clonogenicity assay for cisplatin in nontargeting or β‐TrCP‐silenced HT1376 cells. They were treated with 5 μm cisplatin for 48 h. Histograms show the number of colonies formed after 15 days. Data are presented as mean ± SD (All experiments were replicated three times).

To confirm our hypothesis, β‐TrCP was transiently silenced by siRNA in the parental HT1376 cells. First, we checked the silencing of this protein through mRNA levels by RT‐qPCR (Fig. 2A) and protein levels by western blot (Fig. 2B), and the increase in its substrates by western blot (Fig. 2C and Fig. S2A). After the treatment with 5 μm cisplatin for 48 h, we observed that the levels of the apoptotic markers cleaved PARP and caspase‐3 diminished as compared with siRNA control. The same results were obtained after silencing β‐TrCP in the wild‐type β‐TrCP‐overexpressing cells (Fig. 2D), which was also concordant with apoptosis analysis by Annexin V assay (Fig. S2B), leading us to conclude that the downregulation of the protein conferred cisplatin resistance. Furthermore, to demonstrate that overexpression of β‐TrCP does not induce changes in the cell cycle, we analyzed by flow cytometry the percentage of cells in each phase of the cycle in U2OS cells after modifying β‐TrCP levels, as well as in HT1376 and HT1376::HA‐β‐TrCP cells after β‐TrCP silencing (Fig. S2C,D). Also, to further demonstrate that the depletion causes cisplatin resistance, we performed a clonogenic assay in the parental HT1376 cells, showing an increased number of colonies in β‐TrCP‐silenced cells compared with the nonsilenced controls (Fig. 2E).

### 3.3 Overexpression of β‐TrCP delays DNA damage repair and enhances cisplatin response

To better understand the relationship between β‐TrCP and cisplatin response, we studied the DNA damage induced by cisplatin and, for that, we treated the cells with 5 μm of cisplatin for 1 h, and then, it was removed, analyzing the DNA repair through RAD51 and γH2AX^Ser139^ signal over time by western blot and immunofluorescence. Furthermore, cells were previously synchronized in the G0/G1 phase by deprivation of serum for 16 h, and the experiment was performed in the presence of 20 μm Z‐VAD‐FMK pan‐caspase inhibitor to prevent the γH2AX^Ser139^ antibody from labeling DSBs generated during the replicative stress or the apoptotic response, respectively. Additionally, we verified the synchronization in G1 by flow cytometry analysis of the cell cycle in parental HT1376 cells after 16 h of serum deprivation and confirmed it through the expression levels of PARP and CASP3 in all three cell lines as controls (Fig. S3A,B).

**Fig. 3:**
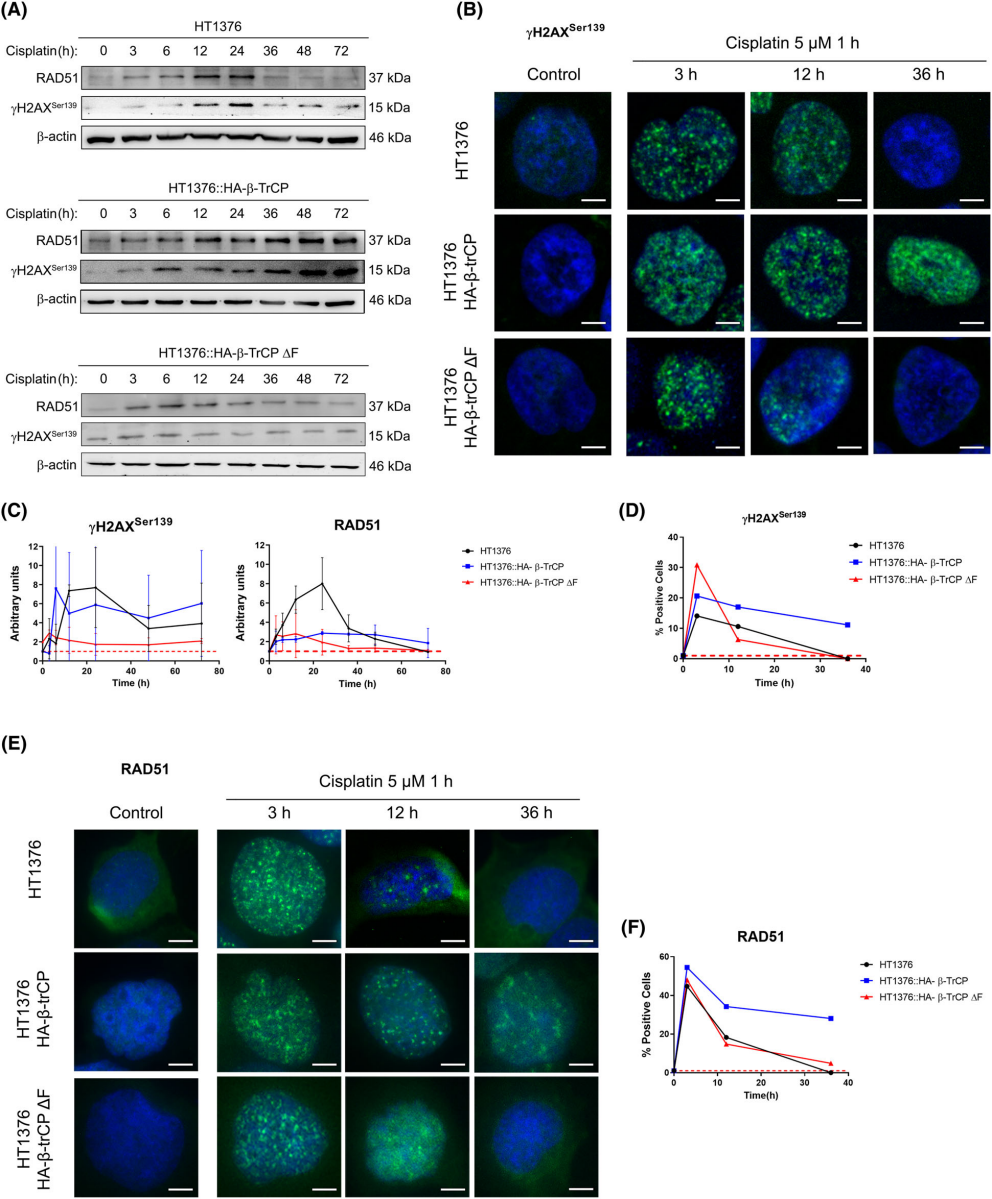
Overexpression of β‐TrCP interferes with the repair of cisplatin‐induced DNA damage. (A) Western blots of RAD51 and γH2AX^Ser139^ in HT1376 (top panel), HT1376::HA‐β‐TrCP (middle panel) and HT1376::HA‐β‐TrCP ΔF (bottom panel) cells at indicated times after the treatment with DMSO (vehicle) or 5 μm cisplatin for 1 h, using β‐actin as a loading control. Cells were previously synchronized in G0/G1 phase by withdrawal of serum for 16 h, and the experiment was performed in the presence of 20 μm ZVAD‐FMK. (B) Immunofluorescence of γγH2AX^Ser139^ in the same conditions. Representative images are shown at the indicated time. The bars represent 20 μm. (C) Quantification of γH2AX^Ser139^ and RAD51 relative levels from (A), normalized to time 0. β‐actin was used as a loading control. (D) Quantification of the percentage of cells positive for γH2AX^Ser139^ from (B), normalized to time 0. A cell was considered positive when its nucleus had at least seven foci. (E) Immunofluorescence of RAD51 in the same conditions. Representative images are shown at the indicated times. The bars represent 20 μm. (F) Quantification of the percentage of cells positive for RAD51 from (E). normalized to time 0. A cell was considered positive when its nucleus had at least seven foci. All experiments were replicated three times.

As we can see in Fig. 3A (top panel), after the treatment in the parental HT1376 cells, RAD51 and γH2AX^Ser139^ levels were gradually induced, with a peak between 12 and 24 h, and then, they progressively disappeared. Indeed, by immunofluorescence we could see γH2AX^Ser139^ foci accumulated at 3 and 12 h, but resolved at 36 h (Fig. 3B, top row). However, when wild‐type β‐TrCP was overexpressed, although cisplatin generated a similar DNA damage, the levels of these proteins remained elevated for longer (Fig. 3A, middle panel), so the γH2AX^Ser139^ foci could still be observed at 36 h (Fig. 3B, middle row). Interestingly, with the overexpression of the mutant β‐TrCP ΔF, there was a modest accumulation of γH2AX^Ser139^ at 3 and 6 h, followed by a decline and partial recovery at 72 h. Similarly, RAD51 levels increased up to 24 h but decreased thereafter (Fig. 3A, bottom panel). Also, in the immunofluorescence we could observe γH2AX^Ser139^ at 3 h, which was quickly cleared up within 12 h (Fig. 3B, bottom row). While these dynamics are consistent with the control conditions, the response is noticeably weaker. Quantifications of both proteins are shown in Fig. 3C,D. Similar results were observed with the staining for RAD51 under the same conditions (Fig. 3E). Quantifications are shown in Fig. 3F. Taken together, these results indicated DNA damage repair in the parental HT1376 cells, while there was a delay or an advance in the repair with the overexpression of wild‐type β‐TrCP and mutant β‐TrCP ΔF, respectively, suggesting that β‐TrCP overexpression interfered with DNA damage repair and enhanced cisplatin response.

### 3.4 Overexpression of β‐TrCP associates with depletion of BRCA1 and CtIP

**Fig. 4:**
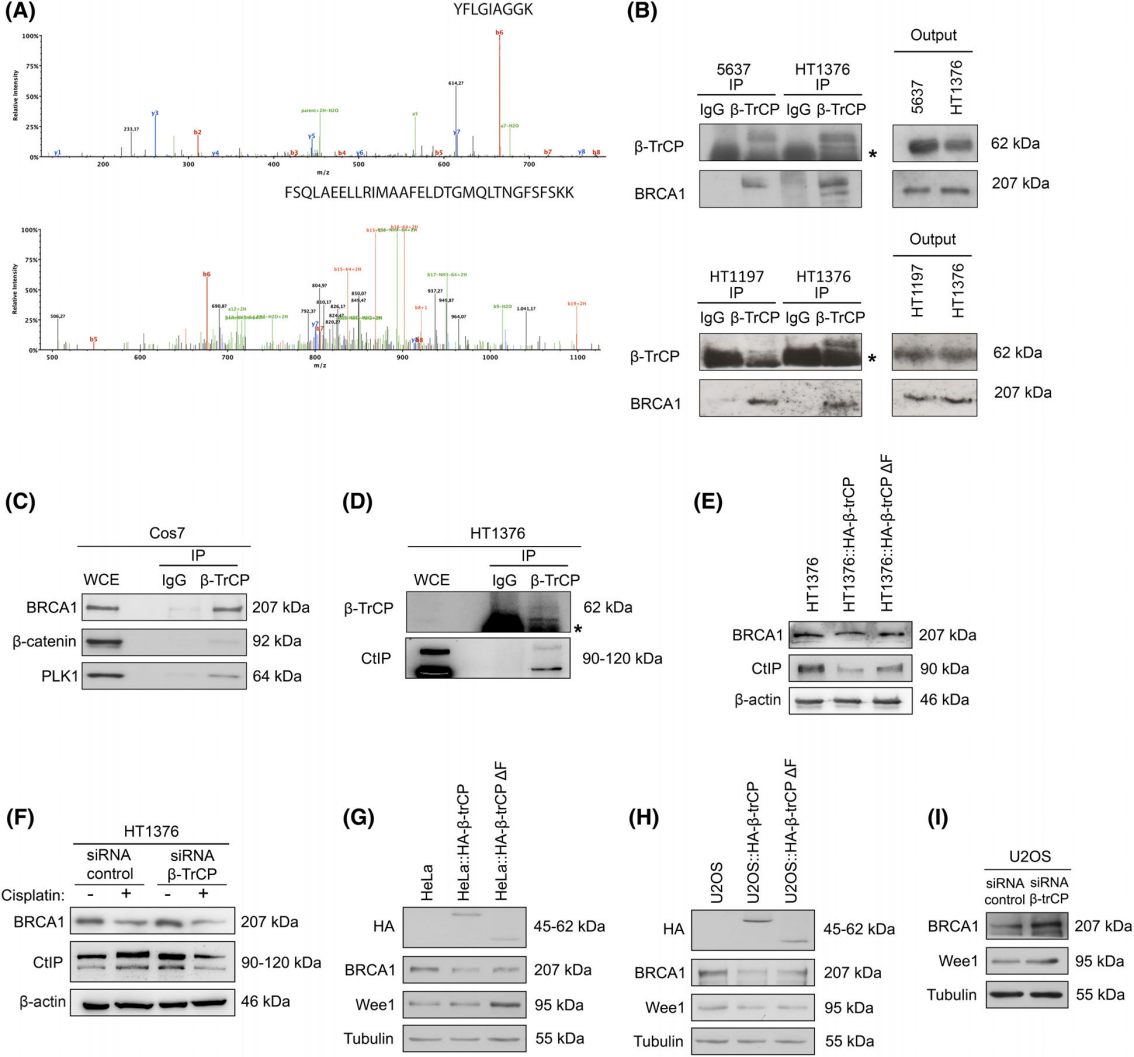
Overexpression of β‐TrCP associates with depletion of BRCA1 and CtIP. (A) Tandem mass spectrometry spectrum of two peptides corresponding to the BRCA1 protein. The diagram shows fragment ions corresponding to the main fragmentation series (β‐amino and γ‐carboxy). (B) Immunoprecipitation assays (IP) of β‐TrCP in 5637, HT1376, and HT1197 cell lines respectively, using total mouse serum (IgG) as control. β‐TrCP and BRCA1 levels were analyzed by western blot. Asterisk represents the expression of IgG. (C) Immunoprecipitation assay (IP) of β‐TrCP in the Cos7 cell line, using total mouse serum (IgG) as control. β‐TrCP, β‐catenin, and PLK1 levels were analyzed by western blot. WCE, Whole‐cell extract. (D) Immunoprecipitation assay (IP) of β‐TrCP in HT1376, using total mouse serum (IgG) as control. β‐TrCP and CtIP levels were analyzed by western blot. WCE, whole‐cell extract. Asterisk represents the expression of IgG. (E) Western blots of BRCA1 and CtIP in HT1376, HT1376::HA‐β‐TrCP, and HT1376::HA‐β‐TrCP ΔF cells, using β‐actin as loading control. (F) Western blots of BRCA1 and CtIP in HT1376 cells transfected with siRNA β‐TrCP or nontargeting siRNA and treated with DMSO (vehicle) or 5 μm cisplatin during 48 h. β‐actin was used as loading control. (G and H) Western blots of hemagglutinin (HA), BRCA1, Wee1, and CtIP in HeLa, HeLa::HA‐β‐TrCP, and HeLa::HA‐β‐TrCP ΔF cells, or U2OS, U2OS::HA‐β‐TrCP, and U2OS::HA‐β‐TrCP ΔF cells, respectively, using tubulin as loading control. (I) Western blots of BRCA1, Wee1, and CtIP in U2OS cells transfected with siRNA β‐TrCP or nontargeting siRNA, using tubulin as loading control. All experiments were replicated three times.

Connecting DNA damage repair and β‐TrCP, we hypothesized that this protein could be regulating the degradation of some repair proteins. To find new SCF(β‐TrCP) substrates, we immunoprecipitated epitope‐tagged β‐TrCP in Cos‐7 cells and identified co‐immunoprecipitating peptides using tandem mass spectrometry. Among the peptides identified, two were unique peptides corresponding to BRCA1 not present in control samples from nonimmune IgG immunoprecipitation (Fig. 4A). To verify the β‐TrCP/BRCA1 interaction, we confirmed the presence of BRCA1 within β‐TrCP immunocomplexes by western blot in 5637, HT1376, and HT1197 bladder cancer cells (Fig. 4B) as well as in Cos7 cells (Fig. 4C). Furthermore, CtIP was also co‐immunoprecipitated efficiently with β‐TrCP in HT1376 cells (Fig. 4D). Next, to demonstrate that this interaction was related to the ubiquitination and degradation of the proteins, we analyzed the levels of BRCA1 and CtIP in the HT1376, HT1376::HA‐β‐TrCP, and HT1376::HA‐β‐TrCP ΔF cells, showing that the levels of both BRCA1 and CtIP decreased significantly in the case of wild‐type β‐TrCP overexpression, with levels of protein similar to wild‐type β‐TrCP after overexpressing the mutant β‐TrCP ΔF (Fig. 4E and Fig. S4A). In addition, this was also verified by silencing β‐TrCP in the parental HT1376 cells where we observed that BRCA1 and CtIP increased as compared with control cells (Fig. 4F and Fig. S4B). However, upon cisplatin treatment, the levels of both BRCA1 and CtIP significantly decrease in both conditions (siRNA β‐TrCP and siRNA control). This reduction is consistent with the known effects of cisplatin on DNA damage response, which leads to the degradation of these repair proteins as part of the damage response mechanism. Finally, the same results were obtained with the overexpression of wild‐type β‐TrCP and mutant β‐TrCP ΔF in different cellular models, such as HeLa (Fig. 4G) and U2OS (Fig. 4H), as well as with the β‐TrCP‐silenced U2OS cells (Fig. 4I). To further confirm that the effect of β‐TrCP on cisplatin response is primarily mediated through BRCA1, we performed BRCA1 knockdown followed by cisplatin treatment. As shown in Fig. S4C, increased cell death after cisplatin treatment was confirmed by elevated levels of the apoptosis markers PARP and caspase 3. We also compared two breast and ovarian cancer cell lines that express different levels of BRCA1. After treating the cells with 5 μm cisplatin for 48 h, we observe that T47D cells, which have high levels of BRCA1, were insensitive to cisplatin treatment, whereas SKOV3 cells, which have low or undetectable levels of BRCA1, are highly sensitive to the treatment (Fig. S4D). This was also supported by a cell viability assay (Fig. S4E). Overall, these results showed that BRCA1 and CtIP depletion was caused by β‐TrCP overexpression, and that this regulation may be crucial for the cisplatin response.

### 3.5 β‐TrCP‐mediated degradation of BRCA1 occurs via both proteasomal and lysosomal pathways

**Fig. 5:**
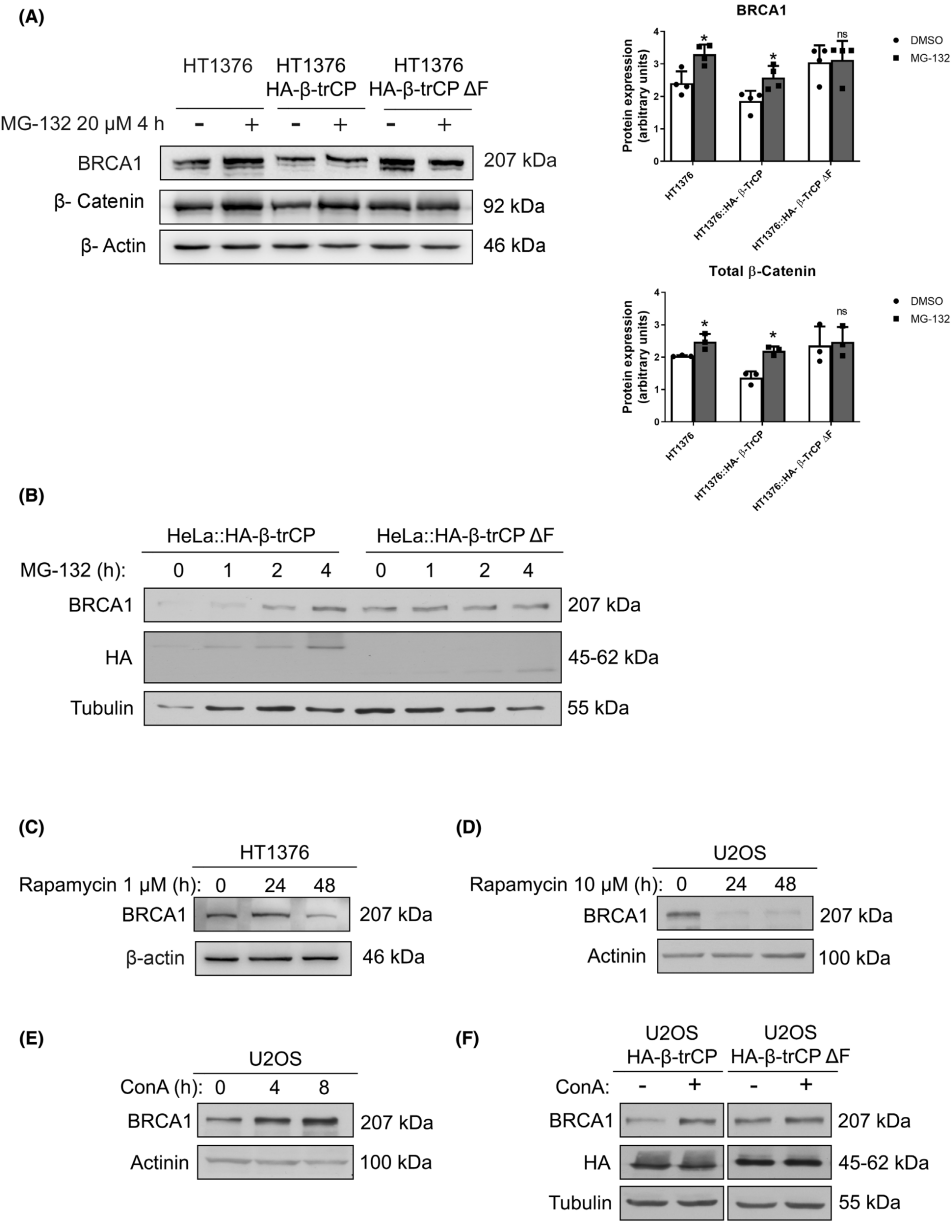
Analysis of degradation pathways of BRCA1. (A) Western blots of BRCA1 and β‐catenin in HT1376, HT1376::HA‐β‐TrCP, and HT1376::HA‐β‐TrCP ΔF cells treated with DMSO (vehicle) or 20 μm MG‐132 for 6 h. β‐actin was used as a loading control. Histograms show the densitometric analysis of the BRCA1 and β‐catenin proteins from the western blot analysis. Data are presented as mean ± SD. *P value < 0.05 from Student's *t*‐test (All experiments were replicated three times). ns, not significant. (B) Western blots of BRCA1 and hemagglutinin (HA) in HeLa::HA‐β‐TrCP and HeLa::HA‐β‐TrCP ΔF cells treated with DMSO (vehicle) or 20 μm MG‐132 for indicated times. Tubulin was used as a loading control. Western blots of BRCA1 in HT1376 (C) and U2OS (D) cells treated with DMSO (vehicle) or rapamycin (1 and 10 μm, respectively) for indicated times, using β‐actin or actinin as loading controls. (E) Western blot of BRCA1 in U2OS cells treated with DMSO (vehicle) or 50 nm concanamycin A (ConA) for indicated times, using actinin as a loading control. (F) Western blots of BRCA1 and HA in U2OS::HA‐β‐TrCP and U2OS::HA‐β‐TrCP ΔF cells treated with DMSO (vehicle) or 50 nm ConA for 8 h, using tubulin as a loading control. All experiments were replicated three times.

BRCA1 degradation has been described as occurring via both the autophagy/lysosome [23] and the proteasome [24], but the potential role of β‐TrCP is not fully addressed. First, HT1376 cells, HT1376 cells overexpressing wild‐type β‐TrCP, or mutant β‐TrCP ΔF were treated with 20 μm of the proteasome inhibitor MG‐132 for 6 h. As we can see in Fig. 5A and the histogram, accumulation of BRCA1 as well as β‐catenin (as a β‐TrCP and MG‐132 control of degradation) occurred after proteasome inhibition in the wild‐type β‐TrCP‐overexpressing cells, while we observed no changes after the treatment with the overexpression of the mutant β‐TrCP ΔF. These results were also more clearly observed in 4 h kinetics of 20 μm MG‐132 treatment in both HeLa cells overexpressing wild‐type β‐TrCP and mutant β‐TrCP ΔF (Fig. 5B), suggesting that β‐TrCP mediated proteasome‐dependent BRCA1 degradation. Next, the autophagy modulators rapamycin (inducer) and concanamycin A (ConA, inhibitor) were used to find out whether BRCA1 was also being degraded by the lysosomal pathway. The treatment with rapamycin 1 and 10 μm induced a decrease in the levels of BRCA1 after 48 h in HT1376 and U2OS cells, respectively (Fig. 5C,D and Fig. S5A). In contrast, when we treated U2OS cells with 50 nm ConA, there was an increase in BRCA1 levels after 4 and 8 h (Fig. 5E), which could also be observed in wild‐type β‐TrCP‐overexpressing cells but not in mutant β‐TrCP ΔF‐overexpressing cells after 8 h (Fig. 5F). We conducted an analysis of autophagy markers LC3 and p62 following treatment with concanamycin A (ConA) and rapamycin in HT1376 and U2OScells, as controls. As shown in Fig. S5B, in HT1376 cells treated with 1 μm rapamycin, the levels of these proteins increased at 24 h and decreased at 48 h. Conversely, as shown in Fig. S5C, U2OS cells treated with 50 nm ConA for 4 and 8 h exhibited an accumulation of both LC3 and p62 levels, indicating an effective blockade of autophagic flux. This observation is attributed to rapamycin as an autophagy inducer, leading to an upregulation of LC3 and p62 expression associated with autophagy at 24 h. Subsequently, at 48 h, due to the recycling of these proteins through the same autophagic mechanism, their levels decrease, indicating an efficient induction of autophagic flux. Taken together, these results showed that the decrease in BRCA1 levels due to β‐TrCP involved both autophagy and proteasome pathways.

## 4 Discussion

Platinum‐based compounds are currently used to treat approximately half of all patients receiving cancer chemotherapy [1]. However, patients who initially respond well to the treatment become resistant within a short period of time, mainly due to an increase in DNA repair [4]. Therefore, the study of these mechanisms in platinum‐resistant tumor cells can help to develop new strategies to increase sensitivity and prevent cancer progression. Previous contributions have described how SCF(β‐TrCP) or SCF(FBXW7) modulate the response to DNA damage through the degradation of various substrates [25, 26, 27]. In this work, we observed that β‐TrCP may act as a sensitizer in the context of cisplatin response, as its overexpression in the HT1376 bladder cancer cells decreased cell viability and clonogenicity, and enhanced the apoptotic response by inducing increased DNA damage. In contrast, all these effects were reversed when we overexpressed a dominant‐negative mutant form of the protein. Furthermore, we found that in both parental HT1376 and HT1376::HA‐β‐TrCP cells, downregulation of β‐TrCP by siRNA significantly reduced cisplatin‐induced apoptosis, indicating that reduced β‐TrCP levels confer resistance to the treatment. Furthermore, we showed that the function of β‐TrCP in response to cisplatin is related to DSB repair. This was demonstrated by the fact that when we monitored DNA damage repair, we observed that although cisplatin treatment induced a similar RAD51 and γH2AX^Ser139^ signal, their resolution was much slower in HT1376::HA‐β‐TrCP than in HT1376 cells, with nuclear foci of γH2AX^Ser139^ persisting even 36 h after treatment withdrawal. Accordingly, in the case of the HT1376::HA‐β‐TrCP ΔF cells, both RAD51 and γH2AX^Ser139^ signals were lower and γH2AX^Ser139^ foci were rapidly cleared within 12 h of treatment, suggesting that overexpression of β‐TrCP may interfere with DNA damage repair and thereby improve the response to treatment. Our results are supported by previous studies showing that β‐TrCP can act as a tumor suppressor [28, 29] and plays an important role in DNA damage response [30, 31, 32]. A recent study demonstrated that β‐TrCP helps to maintain genomic stability by activating the cell cycle checkpoint and DNA damage repair following genotoxic stress [33]. Similarly, β‐TrCP‐mediated degradation of the AEBP2 oncoprotein appears to enhance cisplatin‐induced apoptosis in ovarian cancer [34]. Conversely, β‐TrCP has also been shown to promote DSB repair and cell survival after chemotherapy [35], so its function appears to be cell context‐dependent. The dual role of β‐TrCP as an oncogene or tumor suppressor reflects its ability to recognize a wide variety of substrates, some promoting tumorigenesis while others counteracting it. Our findings suggest that β‐TrCP acts as a tumor suppressor in the context of cisplatin sensitivity by destabilizing BRCA1 and CtIP, impairing homologous recombination repair. Tumor cells might exploit this mechanism by downregulating β‐TrCP to develop cisplatin resistance. However, these results should be interpreted with caution, as the role of β‐TrCP in cancer biology is highly context‐dependent. More deeply, we have pointed out that the role of SCF(β‐TrCP) after cisplatin treatment is to participate in the regulation of the degradation of HR proteins BRCA1 and CtIP. It has been described that BRCA1 is a substrate of several E3 ligase enzymes that allow its degradation via the proteasome [36, 37, 38], although SCF(β‐TrCP) was not among them [37]. On the other hand, BRCA1 itself possesses E3 ligase activity and ubiquitinates CtIP [39], and given the importance of the interaction between both of them [40, 41], we wanted to test whether CtIP expression was also regulated by β‐TrCP. Our results from the tandem mass spectrometry showed peptides corresponding to BRCA1 in cells transfected with HA‐β‐TrCP, and the β‐TrCP/BRCA1 and β‐TrCP/CtIP interactions were verified in HT1376, HT1197, and Cos‐7 cells. Moreover, overexpression and silencing experiments, as well as the use of inhibitors and activators of protein degradation pathways, clearly demonstrate the involvement of β‐TrCP in the degradation of BRCA1. These results are consistent with other studies showing that BRCA1/2 deficient tumors are less able to repair DSBs via HR and respond better to cisplatin treatment [42], and that secondary mutations that restore BRCA1/2 function favor acquired chemoresistance [43]. As for CtIP, we found no studies describing its relationship with cisplatin resistance, although since it is a key factor regulating the choice of DSB repair pathway through its interaction with BRCA1 [41], it would not be surprising if it were also involved in the treatment response. Similarly, deficiency of other HR proteins, such as XRCC2, has also been shown to be associated with chemotherapy sensitivity [44]. All these observations suggest that HR status has important prognostic and predictive value, at least in specific clinical settings. Furthermore, not only mutations but also expression levels are important, since cells that do not express BRCA1 for other reasons, such as promoter methylation [45] have a similar phenotype. Therefore, studying the regulation of their degradation is becoming increasingly important, which in the case of BRCA1 occurs via both autophagy/lysosome and proteasome pathways [23, 24]. Here, although detailed mechanistic studies would be further required to delineate their specific roles, by using different modulators of these pathways, such as MG‐132, rapamycin, and concanamycin A, we have shown that degradation of BRCA1 mediated by β‐TrCP could be occurring via both autophagy and proteasome pathways, with each of these pathways being predominant depending on the cellular context.

In summary, β‐TrCP overexpression increased cisplatin‐induced DNA damage and apoptosis by interfering with the recruitment of BRCA1 and CtIP. By contrast, the failure of β‐TrCP to promote the degradation of BRCA1 and CtIP enables more active DNA damage repair and the acquisition of resistance to cisplatin treatment. It is expected that strategies to alter the function of these proteins could provide mechanisms to prevent resistance to platinum‐based chemotherapies.

## 5 Conclusions

Our study identifies β‐TrCP as a key modulator of cisplatin sensitivity through its ability to regulate the stability of BRCA1 and CtIP, two critical components of homologous recombination repair. We demonstrate that overexpression of β‐TrCP disrupts DNA damage repair and promotes apoptosis, while its downregulation facilitates repair and contributes to chemoresistance. These findings support a model in which β‐TrCP acts as a tumor suppressor in this context by targeting HR factors for degradation. Given the dual role of β‐TrCP depending on cellular context, future therapeutic strategies aimed at modulating its activity may offer new opportunities to overcome resistance to platinum‐based chemotherapy.

## Conflict of interest

The authors declare no conflict of interest.

## Author contributions

CS and MÁJ conceptualized and designed the study. RJ‐G, AB‐F, MG‐M, LR‐C, BP‐V, and JH‐R performed the experiments and analyzed the data. RJ‐G, FR, CS, and MÁJ supervised the investigation. RJ‐G wrote the draft manuscript. RJ‐G, FR, CS, and MÁJ reviewed and edited the manuscript. All authors read and approved the manuscript.

## Supporting information


**Fig. S1.** β‐TrCP modulates the response to cisplatin treatment. (A, B) Histograms show the densitometric analysis of the indicated proteins from the western blot analysis from Fig. 1A,E respectively. (C) Apoptosis detection by flow cytometry of Annexin V and propidium iodide–labeled cells. HT1376, HT1376::HA‐β‐TrCP and HT1376::HA‐β‐TrCP ΔF cells were treated with DMSO (vehicle) or 5 μm cisplatin for 48 h. Data are presented as mean ± SD. *P value < 0.05 from Student's *t*‐test (All experiments were replicated three times). ns, not significant.

**Fig. S2.** β‐TrCP gene silencing induces cisplatin resistance. (A) Histograms show the densitometric analysis of the indicated proteins from the western blot analysis from Fig. 2C. (B) Apoptosis detection by flow cytometry of Annexin V and propidium iodide–labeled cells. HT1376::HA‐β‐TrCP cells transfected with siRNA β‐TrCP or nontargeting siRNA and treated with DMSO (vehicle) or 5 μm cisplatin during 48 h. Data are presented as mean ± SD. *P value < 0.05 from Student's *t*‐test (*n* ≥ 3). (C, D) Cell cycle analysis of propidium iodide‐stained cells by flow cytometry in (C) U2OS and U2OS::HA‐β‐TrCP cells, and (D) HT1376 and HT1376::HA‐β‐TrCP cells after β‐TrCP silencing for 48 h (All experiments were replicated three times).

**Fig. S3.** Overexpression of β‐TrCP interferes with the repair of cisplatin‐induced DNA damage. (A) Analysis of the cell cycle profile of HT1376 cells. Asinchronic and serum starvation for 16 h are shown. (B) Western blot analysis of active caspase 3 and total PARP proteins using β‐actin as a loading control. HT1376, HA‐β‐TrCP and β‐TrCP‐ΔF cells at indicated times after the treatment with 5 μm cisplatin for 1 h. Cells were previously synchronized in G0/G1 phase by withdrawal of serum for 16 h and the experiment was performed in presence of 20 μm ZVAD‐FMK. (All experiments were replicated three times).

**Fig. S4.** Overexpression of β‐TrCP associates with depletion of BRCA1 and CtIP. (A, B) Histograms show the densitometric analysis of the indicated proteins from the western blot analysis from Fig. 4E,F respectively. Data are presented as mean ± SD. *P value < 0.05 from Student's *t*‐test (*n* ≥ 3). ns, not significant. (C) siRNA BRCA1 in HT1376. Cells were transiently silenced with control siRNA and siRNA BRCA1 and treated with DMSO as control or 5 μm cisplatin for 48 h. Levels of BRCA1, PARP and active caspase 3 were analyzed by western blot using β‐actin as loading control (*n* = 3). (D) Characterization of the response to cisplatin in breast and ovarian cancer cell lines T47D and SKOV3 (*n* = 3). Cells were treated with 5 μm cisplatin for 48 h, using DMSO as a control. The levels of PARP, active caspase 3, BRCA1 and β‐TrCP were analyzed by western blot, using β‐actin as loading control. (E) Cell viability assay for cisplatin in T47D, and SKOV3 cells. Data are presented as mean ± SD. *P value < 0.05 from Student's *t*‐test (All experiments were replicated three times).

**Fig. S5.** Analysis of degradation pathways of BRCA1. (A) Histograms show the densitometric analysis of the indicated proteins from the western blot analysis from Fig. 5C. Data are presented as mean ± SD. *P value < 0.05 from Student's *t*‐test (*n* ≥ 3). ns, not significant. (B, C) Western blot analysis of LC3 and p62 proteins, using β‐Actin as loading control, in (B) HT1376 cells treated with rapamycin 1 μm for 24 and 48 h and in (C) U2OS cells treated with concanamycin A (ConA) 50 nm for 4 and 8 h. (All experiments were replicated three times).

## Data Availability

Data will be provided upon request.
