# Supplementary figures and images for: β‐TrCP overexpression enhances cisplatin sensitivity by depleting BRCA1
Source: Mol Oncol. 2025 Jul 28;20(2):524–40. doi: 10.1002/1878-0261.70089 (PMC12936427; doi:10.1002/1878-0261.70089)

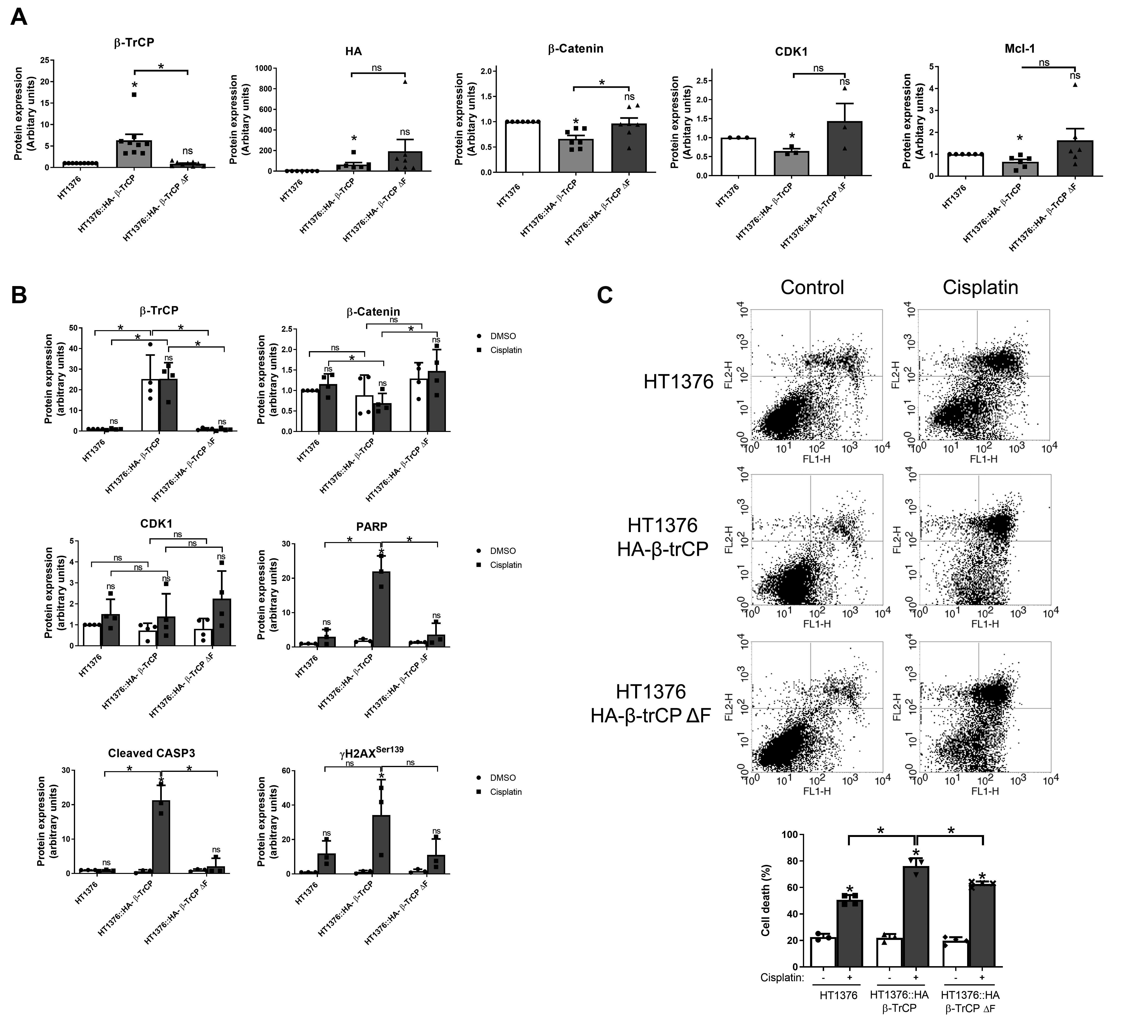

Supplement: Supplementary file 1 — Fig. S1. β‐TrCP modulates the response to cisplatin treatment. (A, B) Histograms show the densitometric analysis of the indicated proteins from the western blot analysis from Fig. 1A,E respectively. (C) Apoptosis detection by flow cytometry of Annexin V and propidium iodide–labeled cells. HT1376, HT1376::HA‐β‐TrCP and HT1376::HA‐β‐TrCP ΔF cells were treated with DMSO (vehicle) or 5 μm cisplatin for 48 h. Data are presented as mean ± SD. *P value < 0.05 from Student's t‐test (All experiments were replicated three times). ns, not significant. [file MOL2-20-524-s003.tif]

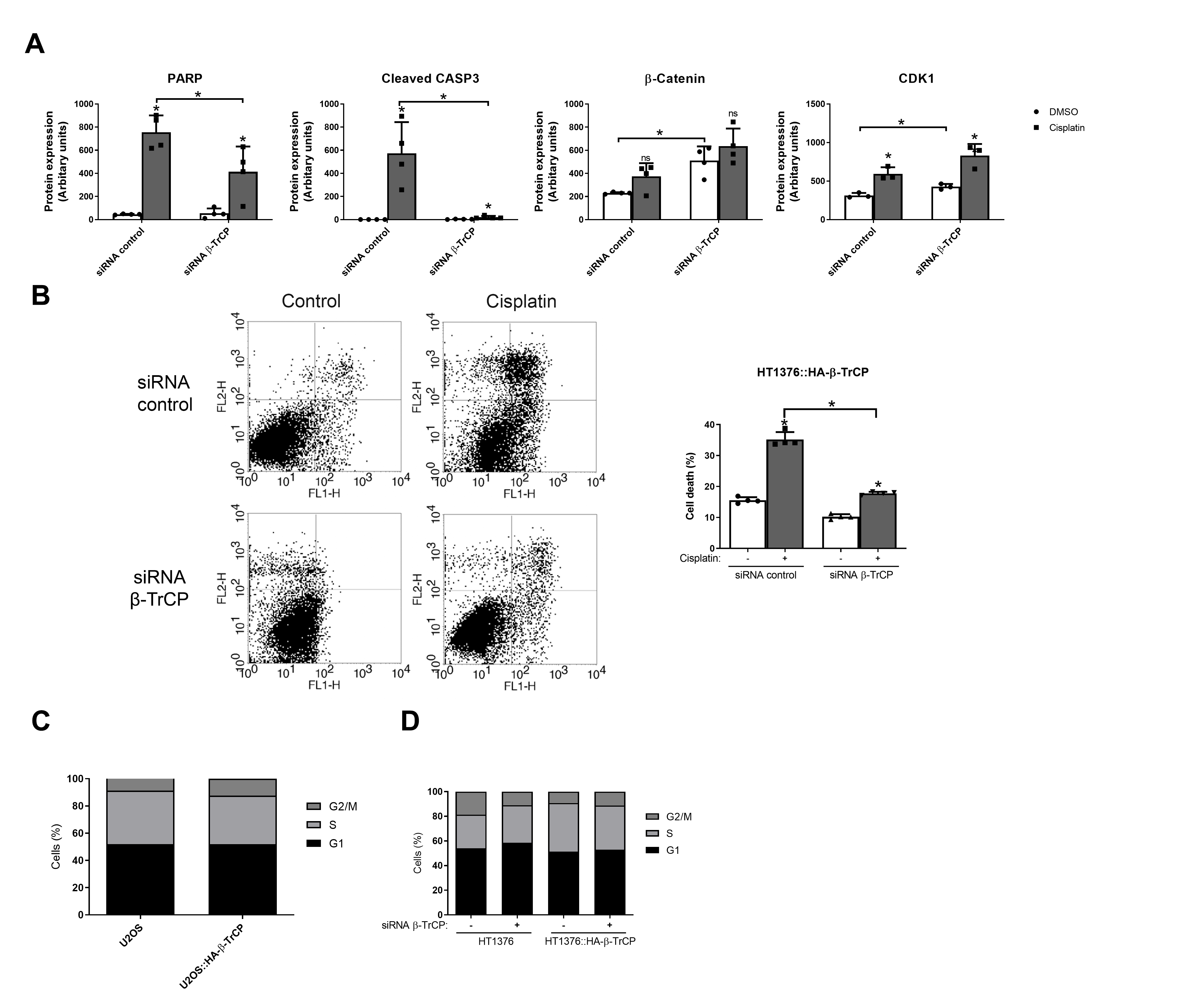

Supplement: Supplementary file 2 — Fig. S2. β‐TrCP gene silencing induces cisplatin resistance. (A) Histograms show the densitometric analysis of the indicated proteins from the western blot analysis from Fig. 2C. (B) Apoptosis detection by flow cytometry of Annexin V and propidium iodide–labeled cells. HT1376::HA‐β‐TrCP cells transfected with siRNA β‐TrCP or nontargeting siRNA and treated with DMSO (vehicle) or 5 μm cisplatin during 48 h. Data are presented as mean ± SD. *P value < 0.05 from Student's t‐test (n ≥ 3). (C, D) Cell cycle analysis of propidium iodide‐stained cells by flow cytometry in (C) U2OS and U2OS::HA‐β‐TrCP cells, and (D) HT1376 and HT1376::HA‐β‐TrCP cells after β‐TrCP silencing for 48 h (All experiments were replicated three times). [file MOL2-20-524-s002.tif]

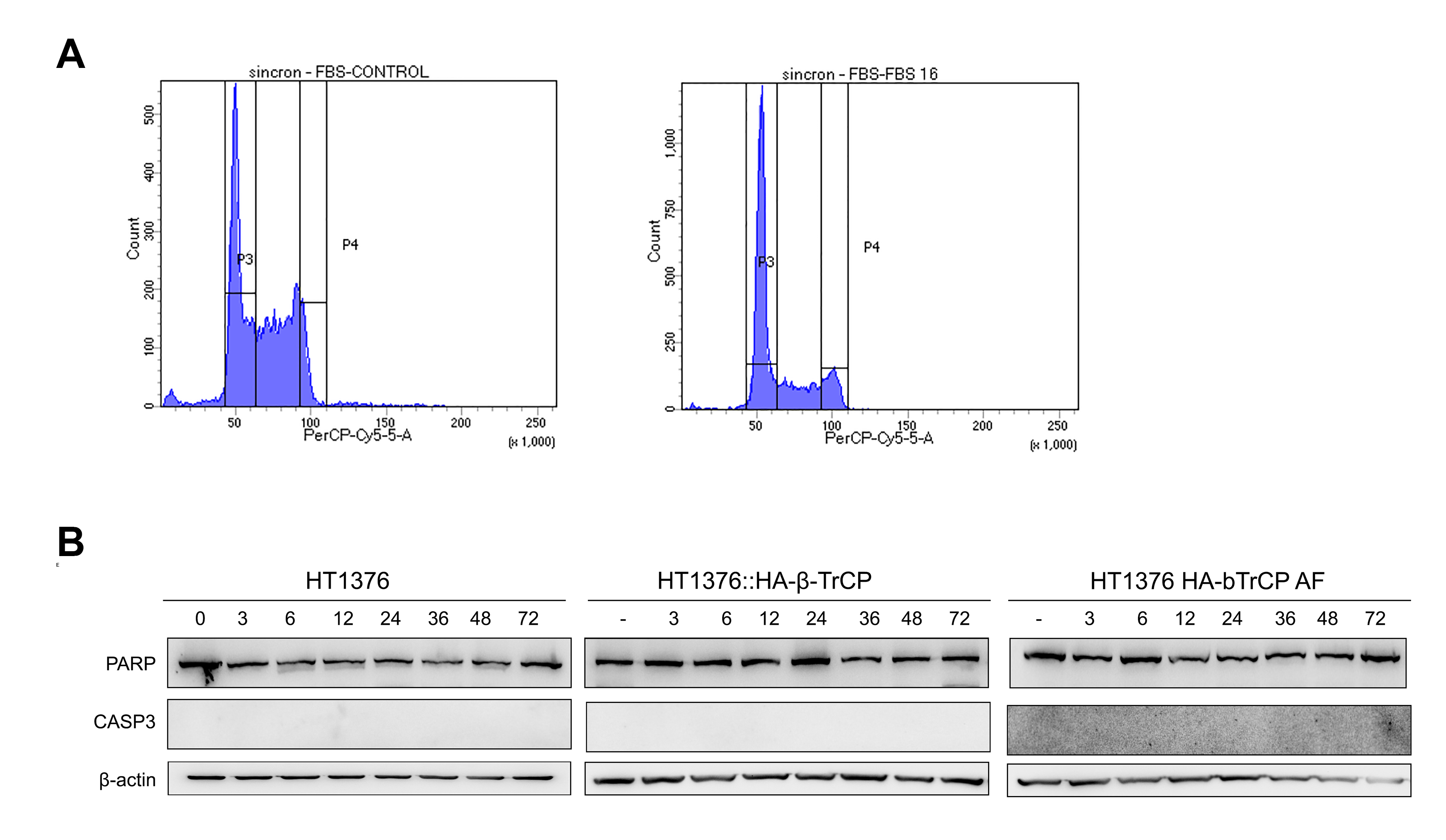

Supplement: Supplementary file 3 — Fig. S3. Overexpression of β‐TrCP interferes with the repair of cisplatin‐induced DNA damage. (A) Analysis of the cell cycle profile of HT1376 cells. Asinchronic and serum starvation for 16 h are shown. (B) Western blot analysis of active caspase 3 and total PARP proteins using β‐actin as a loading control. HT1376, HA‐β‐TrCP and β‐TrCP‐ΔF cells at indicated times after the treatment with 5 μm cisplatin for 1 h. Cells were previously synchronized in G0/G1 phase by withdrawal of serum for 16 h and the experiment was performed in presence of 20 μm ZVAD‐FMK. (All experiments were replicated three times). [file MOL2-20-524-s001.tif]

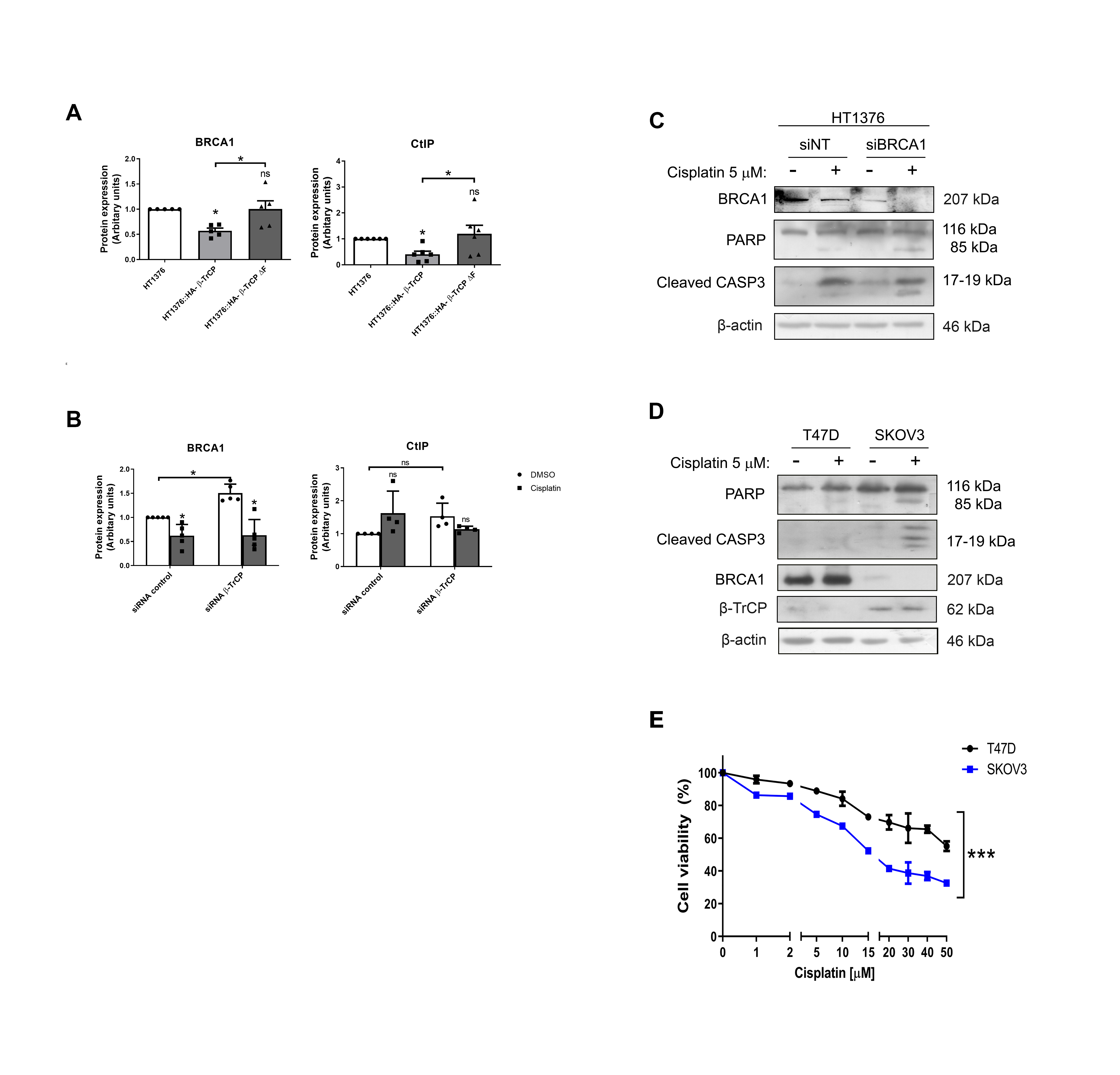

Supplement: Supplementary file 4 — Fig. S4. Overexpression of β‐TrCP associates with depletion of BRCA1 and CtIP. (A, B) Histograms show the densitometric analysis of the indicated proteins from the western blot analysis from Fig. 4E,F respectively. Data are presented as mean ± SD. *P value < 0.05 from Student's t‐test (n ≥ 3). ns, not significant. (C) siRNA BRCA1 in HT1376. Cells were transiently silenced with control siRNA and siRNA BRCA1 and treated with DMSO as control or 5 μm cisplatin for 48 h. Levels of BRCA1, PARP and active caspase 3 were analyzed by western blot using β‐actin as loading control (n = 3). (D) Characterization of the response to cisplatin in breast and ovarian cancer cell lines T47D and SKOV3 (n = 3). Cells were treated with 5 μm cisplatin for 48 h, using DMSO as a control. The levels of PARP, active caspase 3, BRCA1 and β‐TrCP were analyzed by western blot, using β‐actin as loading control. (E) Cell viability assay for cisplatin in T47D, and SKOV3 cells. Data are presented as mean ± SD. *P value < 0.05 from Student's t‐test (All experiments were replicated three times). [file MOL2-20-524-s005.tif]

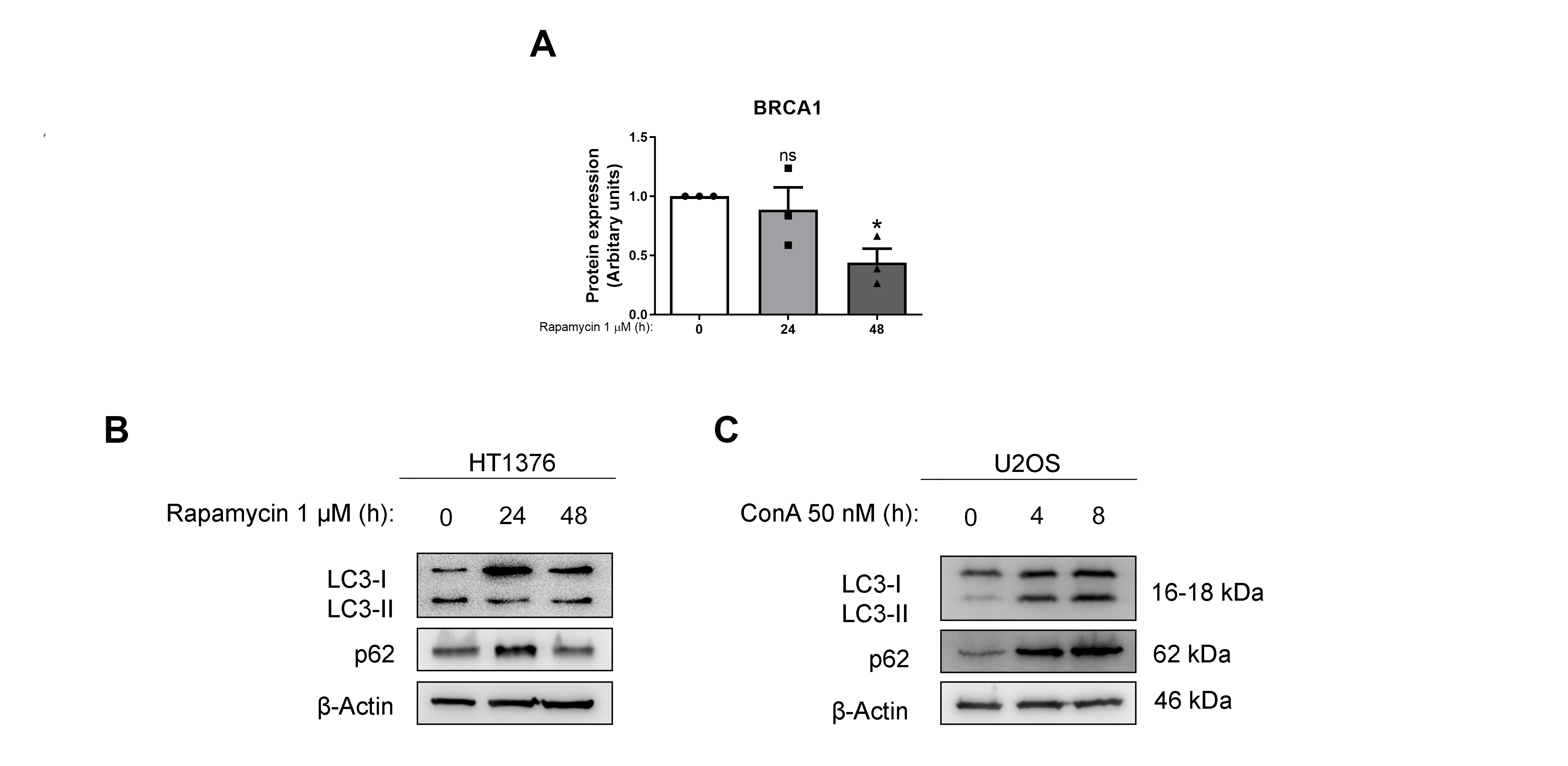

Supplement: Supplementary file 5 — Fig. S5. Analysis of degradation pathways of BRCA1. (A) Histograms show the densitometric analysis of the indicated proteins from the western blot analysis from Fig. 5C. Data are presented as mean ± SD. *P value < 0.05 from Student's t‐test (n ≥ 3). ns, not significant. (B, C) Western blot analysis of LC3 and p62 proteins, using β‐Actin as loading control, in (B) HT1376 cells treated with rapamycin 1 μm for 24 and 48 h and in (C) U2OS cells treated with concanamycin A (ConA) 50 nm for 4 and 8 h. (All experiments were replicated three times). [file MOL2-20-524-s004.tif]
